# New Technologies and Digital Health Tools in Patients With Solid Tumors and Hematological Malignancies: Cross-Sectional Multicenter Survey Study

**DOI:** 10.2196/58823

**Published:** 2026-01-13

**Authors:** Alberto Lopez-Garcia, Carola Diaz-Aizpun, Beatriz Gallego-Diaz, Carolina Miranda-Castillo, Maria Yuste-Platero, Pilar Beltran-Alvarez, Cristina Carames-Sanchez, Jesus Garcia-Foncillas, Pilar Llamas-Sillero, Marta del Olmo-Rodriguez, Jorge Short-Apellaniz, Bernadette Pfang, Javier Arcos-Campillo, Raul Cordoba

**Affiliations:** 1 Lymphoma Unit, Department of Hematology, Fundación Jiménez Díaz University Hospital, Health Research Institute IIS-FJD, Madrid, Spain; 2 Department of Hematology, Regional University Hospital, Malaga, Spain; 3 Department of Hematology, Rey Juan Carlos University Hospital, Madrid, Spain; 4 Department of Hematology, Villalba General University Hospital, Madrid, Spain; 5 Department of Hematology, Infanta Elena University Hospital, Madrid, Spain; 6 Department of Oncology, Fundación Jiménez Díaz University Hospital, Health Research Institute IIS-FJD, Madrid, Spain; 7 Department of Hematology, Fundación Jiménez Díaz University Hospital, Health Research Institute IIS-FJD, Madrid, Spain; 8 Clinical and Organizational Research Unit, Quironsalud Hospital, Avenida Reyes Católicos 2, Madrid, 28040, Spain, 34 900301013

**Keywords:** eHealth, oncohematology, telemedicine, survey, digital health tools, tumor, hematological malignancies, barrier, cancer, cancer care, cross-sectional study, cancer diagnosis

## Abstract

**Background:**

Barriers to eHealth use include lack of technological infrastructure, resistance to change, and inequities in access. However, patterns of access to and use of eHealth tools in people being treated for cancer have not been fully described in the literature.

**Objective:**

Our aim was to describe the patterns of access to and use of eHealth tools among outpatients receiving treatment for hematological malignancies and solid tumors.

**Methods:**

We conducted a cross-sectional multicenter study using a survey offered to patients aged over 18 years receiving outpatient treatment for hematological malignancies or solid tumors at 4 teaching hospitals in Madrid, Spain, between February 1, 2021, and November 30, 2021. The survey instrument featured questions about patients’ demographic and social characteristics, cancer diagnosis, use of information and communication technology (ICT), use and opinions of the Patient Portal, and changes in ICT use during the COVID-19 pandemic. To study the relationship between the different variables, 2-tailed Student *t* tests or ANOVA were used for variables with normal distribution, and the Mann-Whitney or Kruskal-Wallis tests were used for variables with nonnormal distribution. Statistical analyses were performed using SPSS (version 25; IBM Corp) for Windows.

**Results:**

In total, 200 patients were included in our study. Median age was 60 (range 21‐87) years. A total of 130 (65%) patients presented with hematological malignancies. Most (n=181, 90.5%) patients considered that eHealth tools might help to improve communication with the medical team during their treatment. Retired participants (28.6% vs 71.4%; *P*<.001), those older than 60 years (26% vs 74%; *P*<.001), and those without higher education (2.6% vs 97.4%; *P*<.001) showed significantly lower rates of internet use, with no observed sex-related differences. A total of 177 (88.5%) patients found the Patient Portal useful, and 140 (70%) reported increased use of ICT due to the COVID-19 pandemic.

**Conclusions:**

Most (177/200, 88.5%) patients viewed eHealth tools as useful and believed that it was helpful to improve communication with their care team. However, notable gaps in the use of eHealth were observed in certain groups of patients, with significant differences in use due to age, education, and employment status. Strategies to identify subgroups at risk for unequal access to digital health, as well as to facilitate access and use, are warranted.

## Introduction

Using telemedicine to provide care for patients with hematological malignancies has proven to increase access to care and levels of patient satisfaction [1] compared with face-to-face care and leads to cost savings for health care systems. The potential of technology to facilitate the care of immunosuppressed patients, such as those with hematological cancer, has been demonstrated recently during the COVID-19 pandemic [2,3]. During the pandemic, clinical guidelines recommended stopping treatment and performing remote follow-up whenever possible [4], leading to a sharp increase in the use of telemedicine in oncology, with up to 80% of centers using some form of remote care [5].

Despite a sustained increase in telemedicine as a model of care delivery in the postpandemic era, many systems are not adequately prepared for widespread implementation due to a lack of technological infrastructure [6]. Other barriers to the large-scale implementation of telemedicine in the hematology setting include resistance to change from health care providers and patients [7], and unequal access to eHealth among different patient groups based on race, age, and other sociodemographic characteristics [8,9]. Research on patient preferences regarding digital models of health care delivery shows disparate results, with a current lack of evidence as to whether the theoretical benefits of telemedicine translate into real benefits in routine clinical practice [4,5].

Our institution, a hospital network in Madrid, Spain, serving a population of more than 1 million inhabitants, launched an eHealth platform (the Quironsalud Patient Portal) in 2019. The platform allows patients to view appointments and test results, communicate with their health care providers through web dialogues, answer patient-reported outcome measurement (PROM) and patient-reported experience measurement (PREM) surveys, and access other health-related functions such as health education videos. Our study aimed to describe the patterns of access to and use of digital health tools in people with hematological malignancies and solid tumors undergoing outpatient treatment. We also aimed to describe patients’ experiences with the Patient Portal and to report changes in digital health tool use caused by the COVID-19 pandemic.

## Methods

### Study Design

We conducted a cross-sectional, multicenter study, using a survey offered to patients with hematological malignancies or solid tumors receiving outpatient treatment at four teaching hospitals in Madrid, Spain. The primary endpoint of the study was to describe the patterns of access to and use of digital health tools across a sample of outpatients receiving treatment for hematological malignancies and solid tumors. The secondary endpoint was to report patients’ opinions of the Patient Portal and identify changes in the use of digital health tools caused by the COVID-19 pandemic.

### Setting and Participants

Centers included in the study were 4 publicly owned, privately managed hospitals from the Madrid Regional Health System—Fundación Jiménez Díaz University Hospital, Infanta Elena University Hospital, General Villalba University Hospital, and Rey Juan Carlos University Hospital. Patients aged over 18 years with hematological malignancies (lymphoma, myeloma, and acute leukemia) or solid tumors receiving oral or intravenous treatment as outpatients were included. On average, the 4 centers treat 2000 outpatients with cancer per month. We excluded patients who did not undergo active treatment during the study period. Recruitment for the study was conducted between February 1, 2021, and November 30, 2021.

### Variables and Measurements

The survey instrument featured questions about patients’ demographic and social characteristics, cancer diagnosis, use of information and communication technology (ICT), use and opinions of the Patient Portal, and changes in ICT use during the COVID-19 pandemic.

The survey was developed by 2 hematologists (ALG and RC), with survey items developed based on the results of a previous literature review featuring articles on digital health in patients with cancer published in English or Spanish from 2010 to 2020. Survey items were presented as yes or no or multiple-choice questions. Before recruitment began, a pilot questionnaire was administered to 10 patients to validate survey design and comprehension, with no changes made in the original design. The final survey included 30 items divided into 5 thematic blocks, including demographic and social characteristics, cancer diagnosis, ICT use, Patient Portal use, and COVID-19 pandemic–related aspects. Multimedia Appendix 1 shows the items featured in the survey.

All patients who visited the oncology outpatient clinic to receive intravenous treatment during the inclusion period were invited to participate in the study. After informed consent was obtained, participants were handed a paper copy of the survey by the treating physician. The completed surveys were returned in person.

### Bias

We chose to administer a paper-based survey to avoid selection bias caused by the underrepresentation of patients who were not regular ICT users. Illegible or incomplete surveys were excluded.

### Statistical Methods

A descriptive analysis of the population was conducted. Qualitative variables were described using frequency tables. Continuous variables that followed a normal distribution were described by mean and SD. Continuous variables that did not follow a normal distribution were described using the median and IQR.

Normality was verified using the Kolmogorov-Smirnov test. To test for differences in the use of internet and eHealth tools between different groups (in terms of age, sex, and employment status), Student *t* tests or ANOVA were used for variables with normal distribution, and the Mann-Whitney or Kruskal-Wallis tests were used for variables with nonnormal distribution. Statistical analyses were performed using SPSS (version 25; IBM Corp) for Windows.

### Ethical Considerations

This study was conducted in accordance with the ethical standards of the institutional research committee, Hospital Clinical Research Ethics Committee (ER_PIC018-20_FJD), and the Declaration of Helsinki. All participants provided written informed consent before participating in the study. All data were deidentified before performing statistical analysis, and the privacy and confidentiality of the research participants’ data were maintained throughout the study. No compensation was offered to participants or researchers.

## Results

A total of 225 questionnaires were received, with 200 (88.9%) questionnaires included in the final analysis (approximately 0.1% of the total number of eligible patients). Reasons for exclusion from the final analysis included incomplete surveys (11/25, 44%), having marked multiple options on a single-response question (4/25, 16%), and failure to meet eligibility criteria in terms of age (10/25, 40%). Demographic, clinical, and social characteristics of participants and their use of ICT are summarized in Table 1. The median age of the participants was 60 (range 21‐87) years, and 119 (59.5%) participants were male. More than half (n=116, 58%) of the participants had attended higher education institutions. Regarding employment status, 84 (42%) were actively working and 84 (42%) were retired. Most patients had hematological malignancies (n=130, 65%; Table 1).

Notably, 172 (86%) of the 200 patients owned smartphones, and 81 (40.5%) participants owned wearable devices. Regarding reported use of ICT, 136 (68%) patients reported daily use of the internet, and 85 (42.5%) patients conducted health-related research via the internet. Patients older than 60 years (26% vs 74%; *P*<.001; odds ratio [OR] 1.35, 95% CI 1.21-1.51), without higher education (2.6% vs 97.4%; *P*<.001; OR 11.05, 95% CI 3.45‐35.48) and retired (28.6% vs 71.4%; *P*<.001; OR 1.36, 95% CI 1.19-1.57) reported higher rates of never using the internet for browsing (Table 1). However, all patients used social media. Most (n=181, 90.5%) patients considered that eHealth tools might help to improve communication with the medical team during their treatment. The preferred way to communicate with the clinical team was via a smartphone via a WhatsApp-like chat (n=90, 45%).

Regarding the use of eHealth tools, no sex differences were found. Factors associated with higher rates of health-related research via the internet included being over 60 years old (67.3% vs 32.7%; *P*=.02; OR 2.43, 95% CI 1.37‐4.34) and having higher education (44.8% vs 55.2%; *P*<.001; OR 1.65, 95% CI 1.3‐2.1). In contrast, employment status was not significantly associated with the use of the internet to conduct health-related research.

Regarding use of the Patient Portal, we found that patients over 60 years (86.5% vs 13.5%; *P*=.009; OR 4.82, 95% CI 1.34‐17.35) and without higher education (85.7% vs 14.3%; *P*=.01; OR 3.31, 95% CI 1.21‐9.05) had lower Patient Portal registration rates (Table 2). Regardless of registration status, patients aged more than 60 years (78.8% vs 21.2%; *P*<.001), without higher education (94.8% vs 5.2%; *P*=.002), and with an employment status of “unemployed” or “retired” (*P*<.001), preferred not to use the Patient Portal to communicate with their care team (Table 3). Regarding the perceived usefulness of the Patient Portal, more than 80% (177/200, 88.5%) of patients reported the portal’s PROMs and PREMs questionnaires as “useful.” No significant differences were found between groups except regarding employment status, with almost 20% of retired patients considering the PROMs and PREMs questionnaires as “not useful” (Table 4).

When asked whether the use of ICT could improve the patient-clinician relationship, patients’ answers varied. Subgroups tending to consider that ICT could worsen their relationship with the clinical team included patients over 60 years of age and retired patients. Between 20% (40/200) and 40% (80/200) of patients across all groups considered that the use of ICT did not affect their relationship with their care team. Finally, regarding the COVID-19 pandemic, 140 (70%) patients reported higher rates of ICT use. A total of 120 (60%) patients reported no perceived changes in their relationship with the care team, 60 (30%) patients reported an improvement, and 20 (10%) patients reported a decline in their relationship with the health care professional.

**Table 1.** Characteristics of the study population (N=200) and use of information and communication technologies.

Characteristics	Values
Age (y), median (range)	60 (21‐87)
Sex, n (%)	
Male	119 (59.5)
Female	81 (40.5)
Tumor type, n (%)	
Lymphoma	86 (43)
Multiple myeloma	30 (15)
Others	17 (8.5)
Leukemia	14 (7)
Breast cancer	14 (7)
Lung cancer	14 (7)
Gastrointestinal cancer	11 (5.5)
Prostate cancer	7 (3.5)
Gynecological cancer	4 (2)
Genitourinary cancer	3 (1.5)
Education, n (%)	
No or basic	84 (42)
University or superior	116 (58)
Employment, n (%)	
Student	10 (5)
Active	84 (42)
Unemployed	22 (11)
Retired	84 (42)
Department, n (%)	
Hematology	146 (73)
Oncology	54 (27)
Use of internet, n (%)	
Sometimes or always	173 (86.5)
Smartphone	172 (86)
Wearable	81 (40.5)
Internet access, n (%)	
Computer	118 (59)
Tablet	68 (34)
Smartphone	166 (83)
Health research on the internet	85 (42.5)
Preference to communicate with medical team, n (%)	
Smartphone	90 (45)
Email	42 (21)
App	26 (13)
Video call	24 (12)

**Table 2.** Demographic characteristics of patients and their correlation with Patient Portal use.

	Yes, n (%)	No, n (%)	*P* value	Odds ratio (95% CI)
Age (y)			.009	4.82 (1.34-17.35)
<60	93 (96.9)	3 (3.1)		
>60	90 (86.5)	14 (13.5)		
Education			.01	3.31 (1.21-9.05)
No or basic	72 (85.7)	12 (14.3)		
University or superior	111 (95.7)	5 (4.3)		
Employment			.22	—^a^
Student	10 (100)	0 (0)		
Active	79 (94)	5 (6)		
Unemployed	18 (81.8)	4 (18.2)		
Retired	76 (90.5)	8 (9.5)		

a Not available.

**Table 3.** Patients’ preferences regarding communication with health care professionals via the Patient Portal.

	Yes, n (%)	No, n (%)	*P* value	Odds ratio (95% CI)
Age (y)			<.001	1.04 (1-1.08)
<60	76 (100)	0 (0)		
>60	92 (78.8)	22 (21.2)		
Education			.002	—^a^
No or basic	68 (81)	16 (19)		
University or superior	110 (94.8)	6 (5.2)		
Employment			<.001	—
Student	10 (100)	0 (0)		
Active	82 (97.6)	2 (2.4)		
Unemployed	22 (100)	0 (0)		
Retired	64 (76.2)	20 (23.8)		

a Not available.

**Table 4.** Patients’ perception of the usefulness of the quality-of-life questionnaires.

	Yes, n (%)	No, n (%)	*P* value
Age (y)			.07
≤60	89 (92.7)	7 (7.3)	
>60	88 (84.6)	16 (15.4)	
Education			.54
No or basic	73 (86.9)	11 (13.1)	
University or superior	104 (89.7)	12 (10.3)	
Employment			.03
Student	10 (100)	0 (0)	
Active	78 (92.9)	6 (7.1)	
Unemployed	21 (95.5)	1 (4.5)	
Retired	68 (81)	16 (19)	

## Discussion

### Principal Findings

This paper presents the results of a multicenter cross-sectional study on patients receiving outpatient cancer treatment for hematological malignancies and solid tumors. Our results demonstrate that most (181/200, 90.5%) patients considered that eHealth tools might help to improve communication with the medical team during their treatment. Retired participants (28.6% vs 71.4%; *P*<.001), those older than 60 years (26% vs 74%; *P*<.001), and those without higher education (2.6% vs 97.4%; *P*<.001) showed significantly lower rates of internet use, with no observed sex-related differences. More than 80% (177/200, 88.5%) of the patients found the Patient Portal useful, and 140 (70%) patients reported increased use of ICT due to the COVID-19 pandemic.

### Comparison to Prior Work

Recent studies have demonstrated that eHealth is an effective form of health care delivery in hematology and has been catalyzed by the COVID-19 pandemic. A study focusing on the use of eHealth in patients with cancer [10] reported that patients showed an interest in applications such as managing appointments, obtaining advice about their disease, and communicating with health care professionals. Free access to eHealth tools and medical approval [10,11] are 2 facilitators for eHealth use. However, despite offering potential benefits, unidirectional tools such as SMS text message reminders are not always effective [12], as patient use of ICT predicts the effectiveness of and responsiveness to unidirectional eHealth tools [13]. Clinician reluctance to incorporate eHealth tools due to fears of a possible increase in clinical burden is another barrier to widespread integration [14]. Our study shows that most patients in an outpatient cancer care setting consider that eHealth tools are useful and facilitate communication with their care team during treatment, with the preferred method of communication being a smartphone. Furthermore, almost half (85/200, 42.5%) of the participants conducted health-related research via the internet, highlighting the importance of ensuring access to trustworthy, evidence-based information sources.

Most (140/200, 70%) participants agreed that their use of health-related ICT increased during the COVID-19 pandemic. This finding is consistent with those of many other studies in oncology and other fields of medicine [15-19]. Interestingly, many eHealth initiatives developed or scaled up during the pandemic have now become common clinical practice, with tools such as chatbots gaining increasing recognition as useful resources for patients with cancer [20,21]. While the use of eHealth is increasingly common in cancer care, the effect of ICT on the physician-patient relationship remains unclear. Many studies have concluded that, despite the increasing use of telemedicine, patients still prefer traditional, face-to-face communication over internet-based interactions with clinicians [22,23]. However, other reports suggest that patients value certain aspects of ICT-mediated communication, such as rapid response, easy access, and availability [24]. Our study suggested that most patients did not associate an increased use of eHealth with changes in their relationship with clinicians, while 30% (60/200) of patients reported an improvement, and 10% (20/200) of patients stated that their relationship with clinicians deteriorated with increased eHealth use. Regarding new developments in the field of ICT-mediated health communication, over the last months, generative artificial intelligence–based chatbots using large language models have come to the forefront. However, while critical appraisal of these models regarding cancer-related information demonstrates high accuracy [25], their reliability in the clinical setting has yet to be validated [26], along with important aspects such as ethics, governance, patient preferences, and patient safety [27].

The complexity of oncohematological treatment, the shift toward patient-centered care, and the relevance of PROMs and PREMs in value-based health care make it necessary to introduce new digital health strategies to facilitate the flow of information between health care personnel and the patient. The importance of collecting PROMs and PREMs is growing, and eHealth tools show a high potential as a method for effective, large-scale data collection [28]. However, studies show that response rates are often low, and that the implementation of strategies to promote survey completion can be effective in improving response rates [29]. In our study, most patients perceived the quality-of-life questionnaires as useful. However, further investigation is needed to understand why certain subgroups, such as retired patients, showed lower rates of acceptance regarding PROMs and PREMs surveys.

### Strengths and Limitations

The main strengths of this study include its multicenter nature and relevance to the current health care panorama. Identifying the habits and preferences of patients regarding the use of ICT and health care is essential to guarantee the success of telemedicine-based initiatives [30,31]. Characterizing subgroups of patients who find it difficult to access or adopt new technologies is an important first step toward bridging the digital divide by developing strategies to facilitate access and promote the use of eHealth in these populations [32].

This study has certain limitations. First, the lack of follow-up due to the cross-sectional nature of the study, and the fact that patients receiving inpatient cancer care were not included, were limitations of this study. However, the participation of patients with both hematological malignancies and solid tumors ensured a representative sample of outpatients receiving treatment at the 4 centers. Second, although this study examined patients’ perspectives on eHealth initiatives such as the Patient Portal, it failed to investigate whether eHealth actually increased patient satisfaction, quality of care, or the overall well-being of the patients. Further studies are needed to address this question. Finally, the fact that the third wave of the COVID-19 pandemic occurred during the recruitment period (February 1, 2021-November 30, 2021), leading to heightened restrictions and disrupted delivery of care, could have influenced some of the participants’ answers.

### Conclusions

Technology is transforming the health care sector, including cancer care, where the use of telemedicine to ensure interdisciplinary collaboration and to connect patients with specialist care is widespread. However, notable gaps in the use of eHealth can be observed in certain groups of patients, with significant differences in use due to age, education, and employment status. Strategies to identify subgroups at risk for unequal access to digital health, as well as to facilitate access and use, are warranted.

## Supplementary material

10.2196/58823 Multimedia Appendix 1 Survey items.

10.2196/58823 Checklist 1 STROBE checklist.

## Abbreviations

ICT: information and communication technology
OR: odds ratio
PREM: patient-reported experience measurement
PROM: patient-reported outcome measurement
