# Supplementary material for: New Technologies and Digital Health Tools in Patients With Solid Tumors and Hematological Malignancies: Cross-Sectional Multicenter Survey Study
Source: JMIR Cancer. 2026 Jan 13;12:e58823. doi: 10.2196/58823 (PMC12798843; doi:10.2196/58823)
Supplement: Multimedia Appendix 1 [file cancer-v12-e58823-s001.docx]

| **Variables included in the Survey** |
| --- |
| **Demographic and social characteristics** |
| - Age: years |
| - Sex: Male/ Female |
| - Education: No studies / Primary education / Secondary education / non-university education / University education |
| - Employment status: Student / Active / Active / Unemployed / Retired |
| - Marital status: Single / Married / Married / Separated / Widowed |
|  |
| **Tumor characteristics** |
| - Service where you are seen: Hematology / Oncology |
| - Where you complete the questionnaire? Outpatient / Day hospital |
| - Type of tumor: Lymphoma / Myeloma / Leukemia / Breast / Lung / Prostate / Digestive / Genitourinary / Gynecologic / Others |
| - Route of treatment administration: Oral / Intravenous or subcutaneous / Both |
| - Type of treatment: chemotherapy / immunotherapy / targeted oral therapies |
| - Line of treatment: 1st line / Relapse |
|  |
| **Characteristics of Information and Communication Technologies (ICTs) management** |
| - Do you consult the Internet in your daily life? Every day / More than 1 time a week / Never. |
| - Do you have a Smartphone or smart phone with internet access: Yes/ No |
| - Do you have Smartwatch or smartwatch with internet access or any other wristband type device that monitors activity (e.g., steps, heart rate,): Yes/No |
| - What device do you use to access the internet (multiple choices): Computer / Tablet / Mobile phone. |
| - Do you consult the internet to solve doubts about your disease? Yes/No |
| - Do you tell your doctor about aspects of your illness that you consult on the internet: Always / Sometimes / Never |
| - Do you use email? Yes/No |
| - Which social networks do you normally use? (Several options): WhatsApp/ Facebook / Instagram / Twitter / Blog / Other |
| - Do you think that the use of these communication tools could help you during the treatment as a communication between the patient and the team (doctor, nurse, pharmacist) that takes care of her? Yes/No |
| - On which device would you prefer to use it (multiple choices): Computer/ Tablet / Mobile |
|  |
| **Characteristics of the use of the Patient Portal** |
| - Do you know what the Patient Portal is? Yes/No |
| - Are you registered in the Patient Portal? Yes/No |
| - Through what device do you access the patient portal? Yes/No |
| - What do you typically use the patient portal for? (Multiple choices): View test results ordered by my doctor / View reports / View and manage appointments / View health information and recommendations |
| - Would you like to be able to communicate with your care team (doctor, nurse, pharmacist) through the patient portal? Yes/No |
| - Do you think it would be useful for you and would help your medical team to follow you better if quality of life questionnaires and early detection of symptoms were sent to you through the patient portal? Yes/No |
| - Do you think that the relationship with your medical team could improve or worsen through the portal: Could improve / Could worsen / No change |
|  |
| **Characteristics of the use of new technologies due to the pandemic of COVID-19** |
| - Do you think the COVID-19 pandemic has increased your use of new technologies to communicate with your medical team? Increased / Decreased / No change |
| - Do you think that the relationship with your medical team has changed with the COVID-19 pandemic: Improved / Worsened / Unchanged |
